# Molecular-Guided Therapy for Melanoma in Canada: Overview of Current Practices and Recommendations

**DOI:** 10.1177/12034754241303057

**Published:** 2024-12-11

**Authors:** Ahmed Shah, Ryan Decoste, Kaitlin Vanderbeck, Anurag Sharma, Simon F. Roy, Karen Naert, Allison Osmond

**Affiliations:** 1 Department of Pathology and Laboratory Medicine, University of Calgary, Calgary, AB, Canada; 2 Alberta Precision Laboratories, Calgary, AB, Canada; 3 Department of Laboratory Medicine and Pathology, Mayo Clinic, Rochester, MN, USA; 4 Department of Pathology, Nova Scotia Health (Central Zone) and Dalhousie University, Halifax, NS, Canada; 5 Department of Laboratory Medicine and Pathobiology, University of Toronto, Toronto, ON, Canada; 6 Pathology and Laboratory Medicine, Western University, London, ON, Canada; 7 Department of Dermatology, Yale University, New Haven, CT, USA; 8 Department of Diagnostic and Molecular Pathology, Memorial University of Newfoundland, Health Sciences Centre, St. John’s, NL, Canada

**Keywords:** melanoma, reflex molecular testing, BRAF, biomarkers

## Abstract

The emergence of pathologist-driven molecular reflex testing for tumoural biomarkers is a significant advancement in cancer diagnostics, facilitating targeted cancer therapy for our patients. Based on our experience, the Canadian landscape of pathologist-driven reflex biomarker testing for melanoma lacks standardization and is plagued by a lack of awareness by pathologists and clinicians. This paper comprehensively examines the approaches to reflex biomarker testing for melanoma patients across Canada, highlighting the regional variations in the criteria for initiating molecular testing, the biomarkers tested, and the molecular techniques employed. We also discuss the clinical relevance of biomarkers, emphasizing their alignment with the National Comprehensive Cancer Network^®^ (NCCN^®^) Clinical Practice Guidelines in Oncology (NCCN Guidelines^®^) as well as ancillary tests such as BRAF VE1 immunohistochemistry to detect BRAF V600E mutation and molecular techniques such as real-time polymerase chain reaction, matrix-assisted laser desorption ionization-time of flight mass spectrometry and next-generation sequencing. Our proposed standardized *minimum criteria* for reflex testing prioritize melanomas with Breslow thickness >4 mm or disseminated disease, who will most benefit from enhanced delivery of biomarkers and expedited access to targeted therapies while attempting to balance cost-effectiveness and utilization of public healthcare resources with patient outcomes.

## Introduction

Pathologist-driven molecular reflex testing of tumoural biomarkers is a burgeoning skillset for pathologists, enabling the identification of tumoural molecular drivers, and facilitating targeted cancer therapy for patients. A pathologist-driven approach reduces turnaround times for biomarker results and may allow for quicker initiation of cancer therapy when compared to retroactive oncology-driven testing strategies. Particularly in non-small cell lung cancer (NSCLC), where testing for biomarkers is widely practiced using either immunohistochemistry (IHC) or next-generation sequencing (NGS), this method has demonstrated significant efficacy.^1-3^ Conceptually, reflex testing has expanded to various tumour types, including melanoma, and most notably *B-Raf proto-oncogene* (*BRAF*) mutated melanomas, which exhibit high sensitivity to BRAF- and MEK inhibitors.

Cutaneous melanoma remains a significant contributor to mortality in Canada, with an incidence rate of 20.75 cases per 100,000 individuals diagnosed each year.^4,5^ Recently, a group of Canadian physicians from melanoma-related fields have advocated for the adoption of reflex *BRAF* testing, extending its scope to clinical stage IIB and IIC of melanoma cases.^6^ Their rationale emphasizes the urgency of molecular profiling in these stages to initiate therapeutic interventions promptly, acknowledging the heightened risk for disease progression.

In practice, however, challenges persist in implementing standardized testing protocols across Canada for melanoma patients due to several factors, including: (1) the geographically dispersed nature of dermatopathologists and general practice pathologists across the country, (2) funding limitations, (3) variations in testing availability, and (4) lack of awareness and adherence to recommendations. Interpreting the complex milieu of molecular assays poses additional challenges for both pathologists and oncologists, complicating the implementation process. All of these complexities exacerbate the existing disparities in reflex testing practices between provinces for melanoma patients.

To address these disparities, it is imperative to first gain a comprehensive understanding of the various approaches to reflex biomarker testing across Canada. This paper aims to expound on the diverse practices of reflex biomarker testing for melanoma patients nationwide. By summarizing these practices coast-to-coast-to-coast, we hope to highlight the current landscape of melanoma diagnostics in Canada and highlight potential opportunities for alignment with the current NCCN Guidelines. By proposing standard minimum criteria, we seek to mitigate the disparities in reflex testing, ultimately leading to improved patient outcomes for all melanoma patients irrespective of their location with due consideration given to the efficient utilization of public funds.

## Methods

According to the 2024 National Comprehensive Cancer Network^®^ (NCCN^®^) Clinical Practice Guidelines in Oncology (NCCN Guidelines^®^) for cutaneous melanoma, mutational testing is only recommended for melanomas with at least 1 sentinel node metastasis of greater than 1 mm in size (greater than or equal to pN1a, stage IIIA).^7^ As well, apart from *BRAF*, *KIT*, and *NRAS* testing for cutaneous melanomas, NCCN Guidelines^®^ expresses doubt in the clinical utility of the majority of gene expression profiling tests in prognostication and guiding therapy. Pathologist-driven reflex testing is also not explicitly endorsed by the NCCN in the guidelines.

We therefore conducted an overview via personal communication asking dermatopathologists in tertiary (ie, cancer care) the following information: (1) the presence of reflex testing conducted by pathologists at their institution, (2) the clinical or pathologic stage criteria that warrant reflex testing, (3) the biomarkers tested during reflex testing, and (4) the molecular techniques employed. In provinces where standardized guidelines for reflex testing are already established, such as Ontario’s Cancer Care Ontario guidelines, we consulted these resources for additional information and validation of the gathered data.

## Results

Based on our correspondence, amongst Canadian dermatopathologists, pathologist-driven reflex biomarker testing for melanoma patients in the Canadian *public* healthcare system is varied and exhibits diverse criteria across the provinces, resulting in regional variations in clinical practice and ultimately melanoma care. These protocols are guided predominantly by (1) local dermatopathology and oncology expertise, (2) available resources, and (3) healthcare infrastructure. Even within the provinces, practices from hospital to hospital are institution-dependent and varied (Table 1).

**Table 1.** Current Reflex Testing Practices for Melanoma in Canadian Tertiary Healthcare Institutions.

Province	Criteria	Type of test	Genes
Alberta (Calgary)	>pT4 or positive SLN or distant metastasis	MassArray^®^	*BRAF, NRAS, HRAS, KIT, PTEN, TERT, GNAQ/11*
Alberta (Edmonton)	Upon oncologist request only (not pathologist-driven)	MassArray^®^	*BRAF, NRAS, HRAS, KIT, PTEN, TERT, GNAQ/11*
Atlantic Canada (Nova Scotia)	>pT3b or distant metastasis	IHC followed by next-generation sequencing	IHC: BRAF V600E NGS: *BRAF*, *NRAS*, *KIT*, *GNA11/Q*
British Columbia (Vancouver General Hospital)	Positive SLN or distant metastasis or satellitosis	IHC followed by real-time PCR	IHC: BRAF V600E RT-PCR: *BRAF* (single-gene testing only)
British Columbia (Victoria General Hospital)	Upon oncologist request only (not pathologist-driven)	No data	No data
Manitoba	Upon oncologist request only (not pathologist-driven)	Next-generation sequencing	Large panel including: *BRAF, NRAS, KIT, HRAS, PTEN, TERT, GNAQ/11*
Newfoundland and Labrador	>pT2a melanomas, ulcerated melanomas, positive SLN, distant metastases, in-transit metastatic lesions Acral and mucosal melanomas Melanomas with spitzoid features	Next-generation sequencing	*BRAF, NRAS, KRAS and KIT*
Ontario	>pT3 or positive SLN or distant metastasis	Next-generation sequencing	Large gene panel including *BRAF, NRAS, HRAS, PTEN, TERT, GNAQ/11*
Quebec	>pT3 with high-risk features (ulceration, satellitosis) or distant metastasis NGS where *KIT* mutations are suspected	Real-time PCR Next-generation sequencing	RT-PCR: *BRAF* (single-gene testing only) NGS: at least *KIT* (no regarding other genes included in the panel)
Saskatchewan	>pT4 (>pT2b if age <40) Otherwise, identical to Newfoundland and Labrador’s protocol	Next-generation sequencing	NGS: *NRAS*, *KRAS*, *BRAF*, and *KIT*

Abbreviations: IHC, immunohistochemistry; NGS, next-generation sequencing; RT-PCR, real-time polymerase chain reaction; SLN, sentinel lymph node.

Within Atlantic Canada, particularly Nova Scotia, the criteria for reflex testing by pathologists include: melanomas classified as pT3b (clinical stage IIB-C) and above, those demonstrating metastasis to any tissue, or upon clinician’s request. Testing encompasses upfront BRAF VE1 IHC followed by NGS with a panel including *BRAF*, *NRAS*, *KIT*, and *GNA11/Q*, with consideration for adjuvant therapy in select cases. Cases referred from New Brunswick and Prince Edward Island to Nova Scotia are also subjected to testing based on these criteria. In Newfoundland and Labrador, a comprehensive NGS panel is utilized to test for various activating mutations such as *BRAF*, *NRAS*, *KRAS*, and *KIT*. The criteria for reflex testing by pathologists is expanded in comparison and applies to diverse clinicopathologic scenarios, including newly diagnosed pT2a melanomas (>1 mm in thickness), stage III melanomas with lymph node involvement (sentinel or non-sentinel), in-transit metastatic lesions, and clinically stage IV melanomas with distant metastases. Reflex testing is proposed for all ulcerated melanomas, acral melanomas, mucosal melanomas, and melanomas with spitzoid features as well.

Within Quebec, reflex *BRAF* testing using real-time polymerase chain reaction (RT-PCR) is completed for all melanomas ≥2 mm in thickness, or those presenting with high-risk features such as ulceration or metastases. NGS is also utilized in select cases where *KIT* mutations are suspected (ie, acral or mucosal melanomas).

Ontario’s recommended approach is to test all melanomas with a depth measuring >2 mm (pT3) or metastatic cases. Ontario’s criteria extend to include *KIT* and *NRAS* alongside *BRAF* mutational testing. Furthermore, reflex testing for *GNAQ* and *GNA11* is recommended for all uveal melanomas in Ontario. Practically, it is worth noting that several institutions in Ontario utilize NGS and screen for a larger panel of genetic mutations than the ones recommended by Cancer Care Ontario.

Across the prairie provinces, the approaches are varied. In Saskatchewan, reflex BRAF-only testing is performed for all pT4 (>4 mm in thickness) melanomas, stage III and IV melanomas, and all melanomas with ulceration regardless of thickness. For patients under the age of 40, BRAF-only testing is performed for stage pT2b or higher. Furthermore, *BRAF*-only testing is performed for all recurrent melanomas, acral melanomas, and melanomas with spitzoid features. Whereas, reflex NGS testing with an expanded panel also encompassing *KRAS*, *KIT*, and *NRAS* is only limited to ocular and mucosal melanomas. Within Alberta, the city of Edmonton currently does not practice pathology-driven reflex testing. Whereas, in Calgary, reflex biomarker testing is recommended by pathologists for melanomas classified as pT4 or those presenting with positive lymph nodes or metastasis. This is accomplished by mass array of several specific point mutations in *BRAF*, *KIT*, *NRAS*, *HRAS*, *PTEN*, *GNAQ/11*, and *TERT*. In Manitoba, there is a lack of formal guidelines, resulting in varied reflex testing practices across the province. Molecular testing is retroactive, by oncologists. Based on our survey, some pathologists order *BRAF* testing upfront if deemed clinically relevant and/or anticipated oncologist request(s), while others await requests from oncologists. Negative *BRAF* results prompt further investigation with a 31-gene NGS panel upon oncologists’ request.

In British Columbia (BC) the standard of practice varies between mainland BC and Vancouver Island. At Vancouver General Hospital specifically, reflex testing involves sequential BRAF VE1 immunohistochemistry followed by molecular testing, ideally performed once, upon initial recognition of metastasis (nodal or extra-nodal) or satellitosis and is usually complemented by a single-gene RT-PCR test for confirmation. NGS panels are not performed reflexively but are carried out only on oncologists’ request. In contrast, reflex testing is not pathologist-driven in Victoria.

Finally, the Northern territories send their pathology specimens to provincial centres, where the respective centre-specific protocol determines the standard of care for patients residing within territories. For example, specimens from Nunavut and Northwest territories are sent to Edmonton. However, since Edmonton does not adhere to any pathology-driven reflex testing protocol, patients within these territories are not receiving the same standard of care as those from several provinces listed above. Based on the Laboratory Guide to Services Version 6.0 published in March 2024 by Yukon hospitals, pathology specimens from Yukon (Whitehorse, Dawson City, and Watson Lake) are referred to St. Paul’s Hospital Division of Anatomical Pathology in Vancouver for analysis, and not a tertiary cancer facility, and thus are subjected to the opinions of the pathologists of that institution.

## Discussion

The emergence of pathologist-driven reflex testing for tumour biomarkers directly impacts cancer patient care. A notable example is in NSCLC patients whereby delays in pathologist-driven reflex testing can have dire clinical consequences.^8^ Extending this paradigm to melanoma presents a promising avenue for expediting targeted therapies for melanoma patients and enhancing their survival. Unfortunately, there remain several challenges that impede this process including lack of awareness and lack of funding, that have led to divergent practices in reflex testing across Canada and therefore differential melanoma care based on geographic location.

### Clinically Relevant Biomarkers in Melanoma

In recent years, BRAF- and MEK inhibitors have emerged as a helpful tool in the therapeutic arsenal for melanoma patients. Activating *BRAF* mutations are present in approximately 50% of cutaneous melanomas, and eligible patients receiving a combined BRAF/MEK-inhibitor therapy have a response rate of approximately 70%^9,10^ though these responses are not as durable as the ones observed by immunotherapy. The most common *BRAF* mutations are found in the 600th codon (V600), approximately 4/5th of these are V600E mutations and 1/5th include V600K/R/M/D/G.^11^ *BRAF non-V600* mutations, including *BRAF* fusions, *BRAF* L597, K601, and G469 make up a small minority of remaining *BRAF* activating mutations. Identification of these specific mutations is of clinical significance. Current data indicate that *BRAF* non-V600 mutations generally exhibit a poor overall response to BRAF inhibitor monotherapy.^12^ However, combined BRAF/MEK inhibition could be considered for these mutations.^13^ The clinical significance of specific *BRAF* mutations is further exemplified by the findings showing that *BRAF* fusions are more sensitive to sorafenib, which is a kinase inhibitor more selective for CRAF than BRAF when compared to specific BRAF-specific inhibitors such as vemurafinib^14,15^ (sorafenib induces apoptosis in melanoma cells with non-V600E mutations^15^).

Primary mucosal and acral melanomas exhibit a lower frequency of activating *BRAF* mutations, with mutations in *KIT* and *NRAS* predominating.^16^ Identification of specific *KIT* gene mutations is essential to determine the sensitivity to KIT-inhibitor therapy (imatinib or nilotinib). Mutations in *KIT* exons 11 and 13 have a high sensitivity to KIT inhibitors than mutations in exon 17 or *KIT* amplifications, which have minimal or no sensitivity.^17-19^ Determining *NRAS* mutations is also of clinical relevance since they correlate with poor survival, and a minority of these mutations have been shown to respond with MEK inhibitors.^20^

Finally, expression of programmed death-ligand 1 (PD-L1) by tumour cells is used as a marker to initiate anti-programmed cell death protein 1 (PD1) therapy in NSCLC. However, routine use of PD-1 inhibitors in unresectable or metastatic melanoma is not guided by PD-L1 expression, and is in fact, not recommended by the NCCN Guidelines.^7^ Although, immune checkpoint therapy has revolutionized the field and dramatically increased survival for patients with metastatic melanoma, several randomized control trials have demonstrated that PD-L1 expression is not predictive of overall survival and progression-free survival in response to immune checkpoint inhibitors such as nivolumab in these patient populations.^21-23^

### Role of Ancillary Techniques in Biomarker Reflex Testing in Melanoma

Various techniques are presently employed to identify clinically actionable melanoma mutations, including IHC at the protein level, mutation-specific RT-PCR, matrix-assisted laser desorption ionization-time of flight mass spectrometry (MALDI-TOF MS), and NGS at the gene level.^24^

Biomarker testing often employs IHC in many institutions, with a typical cost ranging from €22 (approximately 32 CAD) per test.^25^ Currently, the only available IHC antibody for clinical settings is the BRAF VE1 monoclonal antibody, which has a specificity and sensitivity of 98% and 97%, respectively, in detecting *BRAF* V600E mutations in melanoma.^26^ Despite the low cost and the short turnaround time of less than 48 hours, sole reliance on IHC alone for reflex testing is not practical since *BRAF* V600E mutations account for less than half of all cutaneous melanomas. Other practical constraints include variability in interpretation among users. However, it is commonly used in Canada as part of a cost-saving screening strategy to detect *BRAF* V600E mutations.

Finally, the NCCN Guidelines recommends an expanded molecular panel to include other less common clinically relevant genetic targets, including non-*BRAF* V600, *NRAS*, and *KIT* mutations.^7^ In Alberta, MassArray^®^ Dx 4 system (Agena Bioscience Inc., San Diego, CA, USA) is used, which is a type of MALDI-TOF MS platform. This platform runs the iPLEX HS Melanoma panel (Agena Bioscience Inc.), which is a commercially available panel capable of detecting 97-point mutation variants in 11 melanoma genes, which costs approximately $170 CAD per test. In comparison, NGS provides vastly greater genetic information than simple point mutations, and costs approximately €415 (approximately 610 CAD).^27^ Both NGS and MALD-TOF MS platforms have sensitivity and specificity approaching 98% to 100% in detecting *BRAF* mutations in melanoma.^24^ RT-PCR based techniques also have comparable sensitivity and specificity to NGS and MALDI-TOF MS.^24^ Furthermore, each test is able to detect nearly all clinically relevant genetic targets. Therefore, we do not specifically recommend NGS over MALDI-TOF MS and RT-PCR, since apart from identifying mutations to distinguish benign from malignant melanocytic lesions, much of the genetic information provided by NGS may not be clinically actionable *yet*.

### Incorporating Prognostic Factors into the Establishment of Reflex Testing Criteria for Cutaneous Melanoma

Several studies within the USA have proposed nomograms—*statistical models based on multivariate logistic regression analysis that consider interactions of various variables to identify a probability of an event*—for predicting sentinel node metastasis and cutaneous metastasis.^28-30^ The nomogram proposed by Memorial Sloan Kettering Cancer Center is complex and includes multiple variables, including Breslow depth, ulceration, age, and site in its analysis, with a negative predictive value >90%.^28^ However, regarding risk of lymph node metastases, Breslow depth has been independently shown to have the most prognostic significance.^31^ One study analyzing high-risk variables in 348 patients with invasive melanoma, only found Breslow thickness to be most significantly correlated with positive sentinel lymph node status.^32^ Another study analyzing 181 cases of pT3-pT4 melanomas reported that approximately 15% of invasive melanoma cases with a Breslow depth of 3 to 4 mm, and 25% of cases with a depth >4 mm had sentinel lymph node metastases.^33^ Other variables, including ulceration, tumour necrosis, and histologic subtype of melanoma have been explored but there is no consensus as to their validity in prompting reflex testing.^34-40^

### Proposed Standardized Reflex Testing Criteria for Cutaneous Melanoma in Canada

Appreciating that resources are finite in a socialized health care system, we believe the goal for pathologist-driven expedited delivery of biomarkers should prioritize: (1) metastatic disease (lymph node, in-transit metastasis/satellitosis, or to visceral sites) OR (2) localized tumours with high risk for developing metastatic disease. We believe the criteria for reflex testing should take into consideration the evidence regarding when to initiate targeted therapy, the clinical need for expedited care, and finally the availability of resources. An ideal standardized reflex testing criterion is simple, rooted in current clinical practices, and avoids esoteric prognostic variables.

**Figure 1.**
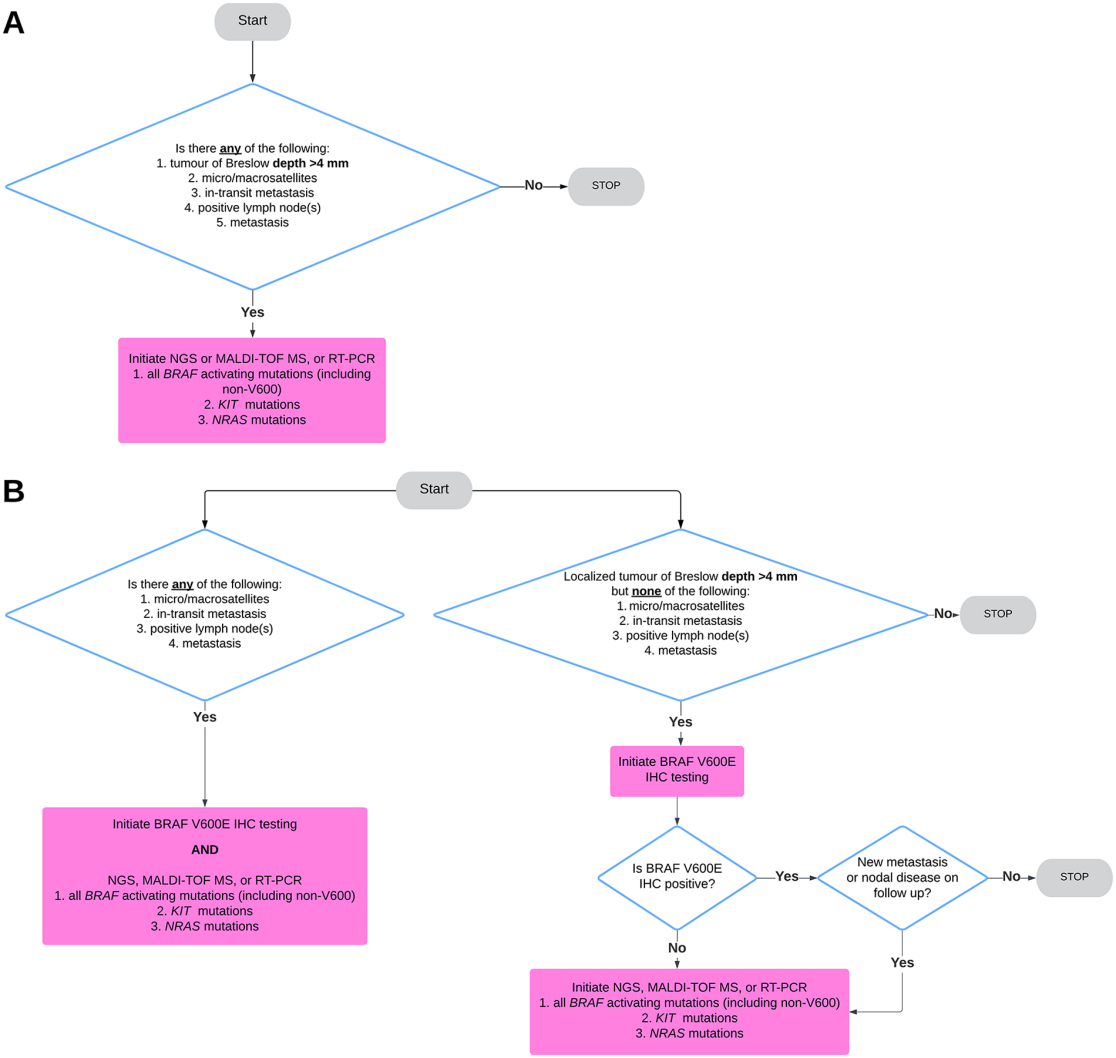
(A) Recommended minimum standard pathology-driven reflex testing algorithm for cutaneous melanoma. (B) Proposed reflex testing algorithm for institutions that wish to proceed stepwise with BRAF V600E immunohistochemistry. However, we advise that for any pN or pM disease molecular testing should be initiated alongside BRAF IHC.

We propose that all cutaneous melanomas with Breslow depth >4 mm regardless of ulceration (pT4a/b), or with disseminated disease (including satellitosis, in-transit metastasis, positive lymph node status, distant cutaneous and extra-cutaneous metastases including occult metastases) be tested for *BRAF* activating mutations (including non-V600), *KIT* and *NRAS* mutations using, RT-PCR, MALDI-TOF MS or NGS (Figure 1). According to our recommendation, a subset of stage IIB and all patients with stage IIC and above qualify for reflex testing.

Recently, Zhou et al advocated reflex testing for stage >IIB melanomas, which includes ulcerated tumours with a Breslow depth of 2 to 4 mm. While acknowledging that there is a heightened risk of progression in pT3b cases on long-term follow-up, the minimal risk of rapid clinical deterioration in localized melanoma, funding limitations, and a turnaround time for NGS averaging 20 days in Canadian laboratories, complicates the justification for reflex molecular testing over testing after oncologic consultation. Nevertheless, we acknowledge the rationale and the potential benefits of extending reflex testing to encompass pT3b melanomas where feasible, such as in Ontario, particularly with advancing technology and cost reductions anticipated in the future. Our aim is to propose standardized minimum criteria for reflex molecular testing across all regions of Canada based on current literature. Keeping Zhou et al team’s recommendation in mind, our recommendation reaches a middle ground by disregarding ulceration as a criterion, and thus incorporation of a subset of stage IIB patients in our simplified reflex testing recommendation for pathologists. The exclusion of ulceration is also important practically speaking for pathologists since determination of *focal* ulceration can be subjective and subject to interobserver disagreement.^38,39^

Although we consider the broad gene panel tests using RT-PCR, MALDI-TOF, and NGS techniques to be the gold standard for reflex testing in melanoma, we acknowledge the cost-effective rationale of several Canadian institutions employing immunohistochemistry using BRAF VE1 monoclonal antibody to assess for *BRAF* V600E mutation prior to initiating molecular testing. As noted previously, half of all cutaneous melanomas harbour activating *BRAF* mutations, of which 80% of them are *BRAF* V600E. With its high specificity and sensitivity, and low turnaround time, BRAF VE1 IHC is a great cost-saving first screening tool for this mutation for localized pT4 tumours without any pN or pM disease (Figure 1B). However, for any pN (satellitosis, in-transit metastatsis, sentinel lymph node metastasis) or pM disease (metastasis to lymph nodes outside the draining basin, visceral or occult metastatic disease), we recommend testing via RT-PCR, NGS, or MALDI-TOF MS to provide oncologists with a larger panel to select potential treatment for aggressive disease. If institutions wish to continue utilizing immunohistochemistry in these instances, we advise incorporating BRAF IHC alongside broad molecular studies. Given its fast turnaround time, BRAF IHC enables the acquisition of potentially relevant therapeutic information while awaiting more extensive genomic profiling, with minimal added costs (Figure 1B). Furthermore, we advocate that any subsequent distant metastases that arise in cases where the initial tumour only received BRAF immunohistochemical testing should be re-tested with a larger molecular panel (Figure 1B).

### Addressing Inequities in Biomarker Testing in Canada

The diversity in reflex molecular testing criteria for melanoma across Canada underscores the need for standardization. This will promote consistent and equitable access to molecular testing for melanoma patients, regardless of geographic location. Much of the available testing discussed above is concentrated in academic (metropolitan) centres in Canada, while many initial melanoma diagnoses are made in community settings that can range from rural areas to smaller cities, where tertiary cancer care centres with melanoma expertise may or may not be located.^41^ The geographic separation between mutation testing centres in Canada and the initial diagnostic centre is very problematic. In fact, transferring melanoma tumour specimen contents to tertiary centres for mutational testing adds to the turnaround time.

This proposal aims to rectify disparities in testing across provinces and territories by promoting the implementation of standardized protocols based on current NCCN Guidelines and literature, thus ensuring equitable access to molecular testing for melanoma patients residing in provinces where testing guidelines are currently absent.

## Conclusion

The emergence of pathologist-driven reflex testing for melanoma biomarkers at the time of microscopic evaluation is a robust avenue for expediting tumour-specific and patient-oriented care for melanoma patients. However, an evaluation of the current landscape of melanoma reflex testing in Canada reveals coast-to-coast-to-coast disparities in criteria and awareness, ultimately leading to disparities in patient care. We highlight several regions, especially Northern territories, that are not meeting the standards we are advocating. With standardization guided by clinical urgency, the most relevant clinicopathologic prognostic factors, and resource availability, pathologist handling of melanoma tumour specimens can be optimized, further improving melanoma patient care. Our proposed minimum standard reflex testing criteria, focusing on high-risk tumours with a thickness of >4 mm or disseminated disease, should streamline testing protocols and enhance equitable access to targeted therapies.
